# Correction: Addressing vaccine hesitancy in developing countries: Survey and experimental evidence

**DOI:** 10.1371/journal.pone.0307570

**Published:** 2024-07-17

**Authors:** Christopher Hoy, Terence Wood, Ellen Moscoe

There are errors in the Funding statement. The correct Funding statement is as follows: Funding for this study was provided by Gavi, the Vaccine Alliance; the Korea Trust Fund for Economic and Peace-Building Transitions; and The United States Agency for International Development (through the Papua New Guinea UNICEF office). The funders played no role in the study design, data collection and analysis, decision to publish, or preparation of the manuscript. The publication fee has been funded by the Australian and New Zealand Governments through the PPIUF. The views expressed in this publication are the author’s/World Bank’s alone and are not necessarily the views of the Australian and/or New Zealand Governments.

In Survey experiment subsection of Materials and methods, there is an error in the second sentence of the fifth paragraph. The correct sentence is: The survey was approved by the US Department of Health & Human Services, Office for Human Research Protections and data collected in line with World Bank protocols regarding research involving human participants.
