# Supplementary material for: Addressing vaccine hesitancy in developing countries: Survey and experimental evidence
Source: PLoS One. 2022 Nov 17;17(11):e0277493. doi: 10.1371/journal.pone.0277493 (PMC9671457; doi:10.1371/journal.pone.0277493)
Supplement: S1 File — (DOCX) [file pone.0277493.s001.docx]

**S5. Survey weighting**

Both the phone survey and online experiment collected samples of respondents that were broadly representative of the population that had access to their respective communication channels. As a consequence, there were differences in the demographic characteristics (in terms of age, sex and location) between the survey samples and the general population as per the most recent census (1). (See comparisons in table below.) Respondents to both surveys were more likely to be male and younger than the general population and their geographic concentration also differed.

**Characteristics of respondents in survey, experiment and general population**

|  | **Online experiment** | **Phone survey** | **National population** |
| --- | --- | --- | --- |
| Male | 73.2% | 73.3% | 51% |
| Adult population aged 40 years or older | 25.9% | 36.1% | 37.0% |
| Highlands Region | 25.5% | 33.2% | 39.2% |
| Islands Region | 21.6% | 21.2% | 15.1% |
| Momase Region | 41.7% | 29.8% | 25.7% |
| Southern Region | 11.2% | 15.8% | 20.0% |

We calculated inverse probability weights at the individual level for both surveys and applied these weights to adjust for these differences between the survey samples and the general population. This involved determining the frequency of demographic characteristics (in terms of age, sex and location) in the surveys and in the general population from the census. The survey weights reflected these differences. The calculated survey results used are shown in in the tables below.

**Inverse probability weights for phone survey**

|  | **Male** | | **Female** | |
| --- | --- | --- | --- | --- |
|  | **<40yrs** | **>=40yrs** | **<40yrs** | **>=40yrs** |
| **Highlands Region** | 0.810 | 0.842 | 2.136 | 2.221 |
| **Islands Region** | 0.489 | 0.508 | 1.289 | 1.340 |
| **Momase Region** | 0.592 | 0.615 | 1.560 | 1.622 |
| **Southern Region** | 0.868 | 0.903 | 2.290 | 2.381 |

**Inverse probability weights for online experiment**

|  | **Male** | | **Female** | |
| --- | --- | --- | --- | --- |
|  | **<40yrs** | **>=40yrs** | **<40yrs** | **>=40yrs** |
| **Highlands Region** | 0.911 | 1.530 | 2.390 | 4.015 |
| **Islands Region** | 0.414 | 0.696 | 1.087 | 1.826 |
| **Momase Region** | 0.365 | 0.613 | 0.958 | 1.610 |
| **Southern Region** | 1.058 | 1.777 | 2.776 | 4.664 |

**References**

1. Papua New Guinea National Statistics Office. Final Figures - National Population Census 2011. Port Moresby: National Statistics Office; 2013.
