# Supplementary material for: Addressing vaccine hesitancy in developing countries: Survey and experimental evidence
Source: PLoS One. 2022 Nov 17;17(11):e0277493. doi: 10.1371/journal.pone.0277493 (PMC9671457; doi:10.1371/journal.pone.0277493)
Supplement: S2 File — (DOCX) [file pone.0277493.s002.docx]

S6. Survey questions about vaccine hesitancy used in analysis.

Response categories are in italics and within parentheses.

Phone Survey

Do you know if a vaccine for COVID-19 is available in this country?
*(Yes; No.)*

Have you been vaccinated for COVID-19?
*(Yes; No.)*

Are you planning to be vaccinated? 
*(Yes; No; Unsure.)*

Why are you not sure or not planning to be vaccinated?
*(I don't think it will work; I am worried about the side effects; I already had COVID-19; I am not enough at risk of contracting COVID-19; In general, I don’t trust vaccines; It is against my religion; I am worried to get infected with COVID-19 at the health facility; Health facility too far or too hard to get to; It will take too long to get vaccinated/ I don’t have time to get vaccinated; Other (specify).)*

Would you be more likely to receive the COVID-19 vaccine if any of the following individuals/ authorities receive or recommend the vaccine?

*(Family and friends; Religious leaders; Doctors/nurses/pharmacist/health workers; Community leaders; Equivalent of a traditional healer; Scientists and epidemiologists; Celebrities and social media influencers; Other.)*

What is your single most preferred way to receive information about the COVID-19 vaccine?

*(Videos of high-profile people talking about the vaccine; Videos with people who have received the vaccine sharing their experience; Face-to-Face communication with prominent leaders in my community; Church leaders providing correct information during church services; Direct information from health workers; Use people in the community to disseminate information so that their own people can understand; Face-to-Face communication from health care workers; I don't think any of these strategies would work; Other.)*

Do you trust the COVID-19 vaccine?
*(Yes; No; Unsure.)*

To what extent do you agree with the following statement? I believe that most of my family and friends will get the COVID vaccine when they are able to receive it.

*(Strongly Agree; Agree, Neither Agree nor Disagree; Disagree; Strongly Disagree.)*

How do you feel about the possibility that you or someone in your household might become seriously ill from COVID-19 (coronavirus disease)?

*(Very worried; Somewhat worried; Not too worried; Not worried at all.)*

Have you ever received any vaccination, such as against diseases like polio or measles?
*(Yes; No.)*

Online experiment

Do you know if a vaccine for COVID-19 is available in this country?
*(Yes; No.)*

Have you been vaccinated for COVID-19 (at least one dose)?
*(Yes; No.)*

[CONTROL] Do you plan to get the COVID-19 vaccine?

*(Yes; No; Unsure.)*

[EXPERT ADVICE] The COVID vaccines available in PNG are considered safe and highly effective by national and international experts. Do you plan to get the COVID-19 vaccine?

*(Yes; No; Unsure.)*

[SOCIAL NORMS] Papua New Guineans are getting vaccinated against COVID-19! More than 20,000 have done it so far. Help us protect our communities! Do you plan to get the COVID-19 vaccine?

*(Yes; No; Unsure.)*

[RELATIVE SAFETY] COVID-19 vaccines are safe – there have been no severe side effects reported, compared to hundreds of deaths due to COVID-19 in PNG. Do you plan to get the COVID-19 vaccine?

*(Yes; No; Unsure.)*
