# Supplementary figures and images for: Addressing vaccine hesitancy in developing countries: Survey and experimental evidence
Source: PLoS One. 2022 Nov 17;17(11):e0277493. doi: 10.1371/journal.pone.0277493 (PMC9671457; doi:10.1371/journal.pone.0277493)

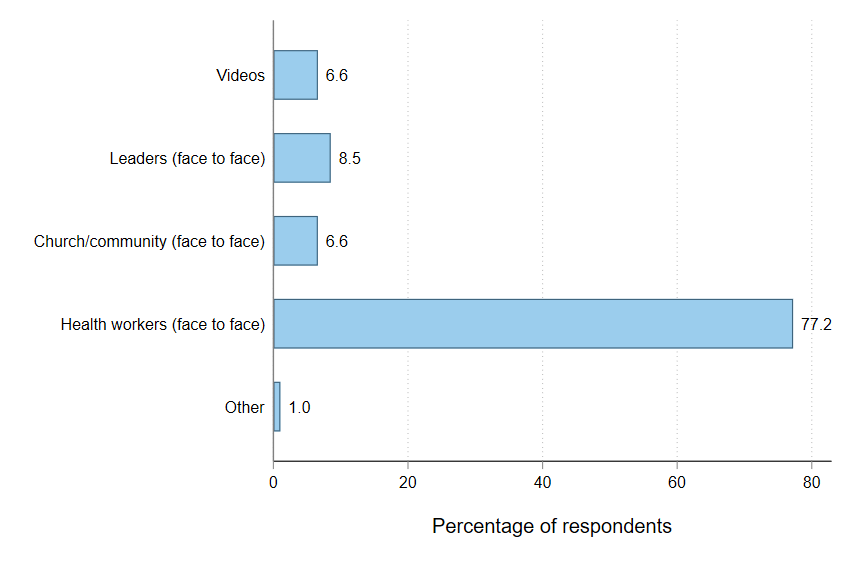


Fig. S1. Preferred mode of vaccine information delivery (from phone survey)

Supplement: S1 Fig — (DOCX) [file pone.0277493.s003.docx]

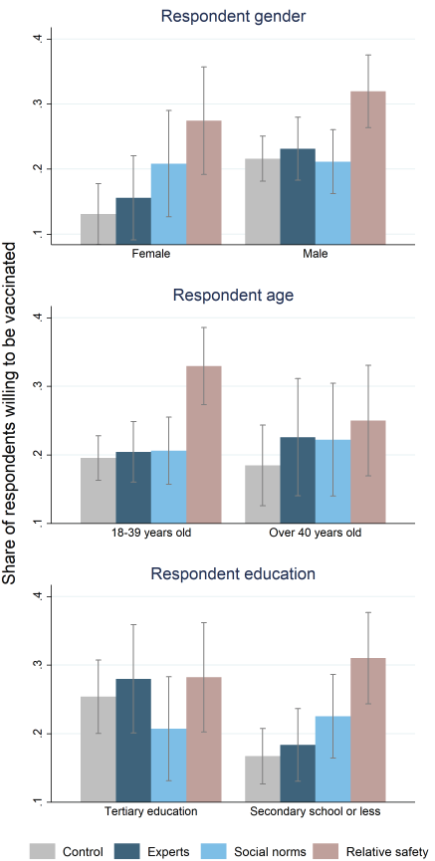


Fig. S2. Experiment treatment effects across demographic groups

Supplement: S2 Fig — (DOCX) [file pone.0277493.s004.docx]
