# Supplementary material for: Addressing vaccine hesitancy in developing countries: Survey and experimental evidence
Source: PLoS One. 2022 Nov 17;17(11):e0277493. doi: 10.1371/journal.pone.0277493 (PMC9671457; doi:10.1371/journal.pone.0277493)
Supplement: S2 Table — (DOCX) [file pone.0277493.s006.docx]

|  | (1) | (2) | (3) | (4) | (5) |
| --- | --- | --- | --- | --- | --- |
| Trust vaccine | -0.51^***^ |  |  |  | -0.43^***^ |
|  | (0.02) |  |  |  | (0.03) |
| Others will be vaccinated |  | -0.39^***^ |  |  | -0.13^***^ |
|  |  | (0.03) |  |  | (0.04) |
| Concerned about COVID |  |  | -0.08^***^ |  | -0.08^***^ |
|  |  |  | (0.03) |  | (0.03) |
| Other vaccinations |  |  |  | -0.04 | -0.03 |
|  |  |  |  | (0.03) | (0.02) |
| Male | -0.06^**^ | -0.08^***^ | -0.09^***^ | -0.09^***^ | -0.06^**^ |
|  | (0.03) | (0.03) | (0.03) | (0.03) | (0.03) |
| Under 35 | -0.01 | -0.02 | -0.02 | -0.01 | -0.02 |
|  | (0.03) | (0.03) | (0.03) | (0.03) | (0.03) |
| Rural | -0.02 | -0.02 | -0.03 | -0.03 | -0.03 |
|  | (0.03) | (0.03) | (0.03) | (0.03) | (0.03) |
| Educated | 0.02 | 0.02 | 0.02 | 0.02 | 0.02 |
|  | (0.03) | (0.03) | (0.03) | (0.03) | (0.03) |
| Wealth | -0.02 | -0.03 | -0.05^*^ | -0.04 | -0.03 |
|  | (0.03) | (0.03) | (0.03) | (0.03) | (0.03) |
| Constant | 0.65^***^ | 0.64^***^ | 0.66^***^ | 0.61^***^ | 0.74^***^ |
|  | (0.04) | (0.04) | (0.04) | (0.04) | (0.04) |
| Observations | 1824 | 1824 | 1824 | 1824 | 1824 |

Dependent variable was binary taking value of 1 if respondent was unwilling to be vaccinated. OLS regressions were used. Robust standard errors in parentheses.

^*^ *p* < 0.1, ^**^ *p* < 0.05, ^***^ *p* < 0.01

**Table S2. Relationship between drivers of vaccine hesitancy and unwillingness to be vaccinated**
