# Supplementary material for: Addressing vaccine hesitancy in developing countries: Survey and experimental evidence
Source: PLoS One. 2022 Nov 17;17(11):e0277493. doi: 10.1371/journal.pone.0277493 (PMC9671457; doi:10.1371/journal.pone.0277493)
Supplement: S3 Table — (DOCX) [file pone.0277493.s007.docx]

|  | (1) | (2) |
| --- | --- | --- |
|  | Willing | Unwilling |
| Experts | 0.03 | 0.01 |
|  | (0.03) | (0.04) |
| Social norms | 0.09^**^ | -0.07^*^ |
|  | (0.04) | (0.04) |
| Relative safety | 0.10^***^ | -0.11^***^ |
|  | (0.03) | (0.04) |
| Male | 0.06^**^ | -0.04 |
|  | (0.03) | (0.03) |
| Over 40 years old | -0.03 | 0.03 |
|  | (0.03) | (0.03) |
| Education | -0.08^***^ | 0.00 |
|  | (0.03) | (0.03) |
| Highlands region | -0.07 | -0.05 |
|  | (0.05) | (0.05) |
| Islands region | -0.13^**^ | 0.02 |
|  | (0.05) | (0.05) |
| Momase region | -0.10^**^ | 0.01 |
|  | (0.05) | (0.05) |
| Constant | 0.28^***^ | 0.29^***^ |
|  | (0.06) | (0.06) |
| Observations | 1514 | 1514 |

Dependent variable was binary taking value of 1 if respondent was willing to be vaccinated (model 1) or
unwilling to be vaccinated (model 2). OLS regressions were used. Robust standard errors in parentheses.

^*^ *p* < 0.1, ^**^ *p* < 0.05, ^***^ *p* < 0.01

Table S3. Treatment effects with sociodemographic controls in regression models
