# Supplementary material for: Addressing vaccine hesitancy in developing countries: Survey and experimental evidence
Source: PLoS One. 2022 Nov 17;17(11):e0277493. doi: 10.1371/journal.pone.0277493 (PMC9671457; doi:10.1371/journal.pone.0277493)
Supplement: S4 Table — (DOCX) [file pone.0277493.s008.docx]

|  | (1) | (2) | (3) | (4) | t-test | t-test | t-test | |
| --- | --- | --- | --- | --- | --- | --- | --- | --- |
|  | Control | Experts | Social norms | Rel safety | diff | diff | diff | |
| Variable | Mean/SE | Mean/SE | Mean/SE | Mean/SE | (1)-(2) | (1)-(3) | (1)-(4) | |
| Male | 0.519 | 0.499 | 0.518 | 0.521 | 0.021 | 0.002 | -0.002 | |
|  | [0.018] | [0.025] | [0.026] | [0.026] |  |  |  | |
| Age (under/over 40) | 0.277 | 0.277 | 0.307 | 0.343 | -0.000 | -0.030 | -0.066** | |
|  | [0.016] | [0.022] | [0.024] | [0.024] |  |  |  | |
| Highlands | 0.388 | 0.367 | 0.383 | 0.326 | 0.021 | 0.005 | 0.063** | |
|  | [0.018] | [0.024] | [0.026] | [0.024] |  |  |  | |
| Islands | 0.154 | 0.178 | 0.152 | 0.200 | -0.024 | 0.001 | -0.046* | |
|  | [0.013] | [0.019] | [0.019] | [0.021] |  |  |  | |
| Momase | 0.276 | 0.263 | 0.253 | 0.313 | 0.012 | 0.022 | -0.038 | |
|  | [0.016] | [0.022] | [0.023] | [0.024] |  |  |  | |
| Southern | 0.182 | 0.192 | 0.211 | 0.161 | -0.009 | -0.029 | 0.021 | |
|  | [0.014] | [0.019] | [0.022] | [0.019] |  |  |  | |
| N | 740 | 415 | 358 | 377 |  |  |  | |
| The value displayed for t-tests are the differences in the means across the groups. | | | | | | | |  |
| ***, **, and * indicate significance at the 1, 5, and 10 percent critical level. | | | | | | | |  |

**Table S4. Balance table across treatment groups in experiment**
