# Supplementary material for: Addressing vaccine hesitancy in developing countries: Survey and experimental evidence
Source: PLoS One. 2022 Nov 17;17(11):e0277493. doi: 10.1371/journal.pone.0277493 (PMC9671457; doi:10.1371/journal.pone.0277493)
Supplement: Attachment — Submitted filename: 2 Response to reviewers.docx [file pone.0277493.s009.docx]

Thank you to the editor and reviewers for their comments on the paper. The comments have been helpful, and have prompted us to improve the paper considerably. Thank you.

All substantive points raised by the editor and reviewer are addressed below. We have addressed the points in the following order: editor, reviewer 1, reviewer 2.

Comments from the editor and reviewers are *in italics*, our responses are in normal text.

**Editor**
*“This paper is topical and both reviewers felt that it had merit.
It did, however, have a number of flaws. The reviewers felt that the introduction required more detail and the description of the actual study design was poor. In addition, there were frequent omissions when it came to abbreviations and statistical packages used etc.”*

Thank you. We have now addressed all unclear abbreviations. We have added full details on the statistical package used as well as other aspects of the methodology. We have done this in a newly-created ‘material and methods section.’

We have addressed all other comments from the reviewers below, and detailed how we have changed the manuscript in response.

**Reviewer #1:** 
*“This interesting manuscript on vaccine hesitancy in Papua New Guinea reports on collected data via two methods: (1) a phone survey of vaccine attitudes and their determinants, followed by (2) an online RCT conducted via Facebook, to measure the differential effects of one of four messages on participants' willingness and unwillingness to be vaccinated. Good care was taken to ensure that the survey and the experiment were representative of the population of Papua New Guinea. The authors are to be congratulated on an extremely well-written paper, devoid of any dense "academese" - it was a pleasure to read such clear writing.

There are a few issues that I feel may strengthen the paper if addressed:

The paper didn't really have a limitations section. Two important limitations are worth mentioning (in my opinion):*

*1. In the survey experiment, while I appreciate that quotas were applied based on demographic characteristics (age, gender and regions), since only 20% of the population use Facebook, the potential of selection bias is still significant. (E.g. perhaps Facebook users are more likely to be open to new experience, or more likely to be susceptible to social messaging, etc.)”*

Thank you for taking the time to review this paper. Thank you for noting this issue, we appreciate that this limitation is worth discussing. We have now created a specific limitations section at the end of the paper. We have now discussed this limitation, alongside the one other limitation you identified, in detail in the newly created section.

*“2. There is a strong potential for the short-term effects seen in the experiment to wane with time. Participants appear to have had to reply almost immediately about their intentions, following different messages. However, we don’t know whether these changes in intentions are durable. Did participants feel the same the next day, or the week after? Or did the effects fade back to baseline? Given that this study can't answer this question (if I'm understanding it correctly), this is a significant potential limitation to the robustness of the findings in the real world.”*

Thank you, this is an excellent point. We have created a specific limitations section at the end of the paper. We have now discussed this limitation, alongside the one other limitation you identified, in detail in the newly created section.

*“The last three paragraphs of the introduction highlight the key results. I'd leave the results for the relevant section though, if possible.”*

Thank you for this suggestion. We have now removed all mention of findings from the introduction.

**Reviewer #2:** 
*“Although the study is relevant, the authors did not research well about the subjects and methodology/tools used to study vaccine hesitancy before writing the manuscripts. The introduction and methodology is poorly written.”*

Thank you for taking the time to review the paper, we have now reformatted it so that the introduction is improved and does not discuss methods and results. We have added a separate and more detailed methods section. We have also added more on the broader literature on vaccine hesitancy to the introduction, citing some of the wide range of papers that informed our research.

*“The introduction is scanty and does not give adequate background of the vaccine hesitancy. It must be re-written and include only introduction and not the method and results.”*

Thank you for this suggestion, we have removed discussion of methods and results from the introduction, and now only discuss these aspects of the paper in the methods and results sections of the paper.

*“Page 3: Paragraph 3 and 4 should move to method and paragraph.
Page 4: paragraph 2-4 should move to results.”*

Thank you for this suggestion, we have removed discussion of methods and results from the introduction, and now only discuss these aspects of the paper in the methods and results sections of the paper.

*“There are no line numbers used in the document and it make it difficult to review- the authors did not follow PLOS instructions guidelines. “*

Thank you for noting this. We have now amended the document, added line numbers, and modified it in other ways to follow PLOS guidelines.

*“Please provide additional details regarding participant consent in the text, if it is written or verbal, and if verbal, how it was documented and witnessed.”*

Thank you for this suggestion, we have now detailed this in the second to last paragraph of the Methods and materials section. (Page 7 in the manuscript without track changes.)

*“Survey Experiment-How did the author ensure that the author ensure that the participant included minors who used wrong age to join Facebook?”*

Thank you for this suggestion, we have now discussed this in the Material and Methods section (in paragraph 1 on page 6 of the manuscript without track changes.) Facebook automatically avoided recruiting people under 18 for our survey based on the date of birth people provided Facebook when they joined. It is true that there is no way of preventing people who lie about their age when signing up to Facebook from participating in online survey experiments. However, we feel that this risk is very low as people have no reason to lie about their age when joining Facebook – people of all ages are allowed to join. To rule out instances where people have mistakenly entered the wrong age when joining Facebook, we double checked by asking people’s ages at the start of the survey and excluded those who stated they were under 18.

*“Statistical analysis: It is not clear which software/program was used for statistics. Please include a section on how the data was analysed and what was considered statistically significance. “*

Thank you for this suggestion. We have now added two sentences on software and statistical significance at the end of methods section. (Paragraph 4 on page 7 in the manuscript without track changes.)

*“The figures need to be correspond to the text eg. Figure 2 does not have any percentages while”*

Thank you, we have now modified those charts currently based on proportions so that they now show percentages, as used in the text.

*“It is not clear which study type was used: cross-sectional survey or convenience sampling etc”*

Thank you for raising this point. The sampling processes for both the survey and experiment are carefully spelt out in the newly-created methods section.

*“It is not clear how was the data collected, managed and captured and whether they used any Likert scale.”*

Thank you for raising this point. The sampling processes for both the survey and experiment are carefully spelt out in the newly-created methods section. All responses were categorical, not Likert, we believe this is clear in the discussion of the responses, and the presentation of the results.

*“The authors need to be consistency if they are using PNG or Papua New Guinea.”*

Thank you for noting this, we have standardised the use of PNG throughout (except in the first instance.)

*“All the abbreviations must be written in full before abbreviation.”*

Thank you. We have now made sure all abbreviations are treated in the appropriate manner.

*“The limitations of the study are not explicitly addressed in the manuscript.”*

Thank you for this suggestion. It is an important point and we have now added a limitations section.
